# Modeling and Design of Chitosan–PCL Bi-Layered Microspheres for Intravitreal Controlled Release

**DOI:** 10.3390/pharmaceutics17091174

**Published:** 2025-09-09

**Authors:** Eduardo A. Chacin Ruiz, Samantha L. Carpenter, Katelyn E. Swindle-Reilly, Ashlee N. Ford Versypt

**Affiliations:** 1 Department of Chemical and Biological Engineering, University at Buffalo, The State University of New York, Buffalo, NY 14260, USA; echacinr@buffalo.edu; 2 School of Mechanical and Aerospace Engineering, Oklahoma State University, Stillwater, OK 74078, USA; s.l.carpenter@ou.edu; 3 Department of Biomedical Engineering, The Ohio State University, Columbus, OH 43210, USA; reilly.198@osu.edu; 4 William G. Lowrie Department of Chemical and Biomolecular Engineering, The Ohio State University, Columbus, OH 43210, USA; 5 Department of Ophthalmology and Visual Sciences, The Ohio State University, Columbus, OH 43210, USA; 6 Department of Biomedical Engineering, University at Buffalo, The State University of New York, Buffalo, NY 14260, USA; 7 Department of Pharmaceutical Sciences, University at Buffalo, The State University of New York, Buffalo, NY 14214, USA; 8 Institute for Artificial Intelligence and Data Science, University at Buffalo, The State University of New York, Buffalo, NY 14260, USA

**Keywords:** polymeric drug delivery, drug release modeling, mechanistic modeling, sustained drug delivery, polycaprolactone, chitosan

## Abstract

**Background/Objectives:** Chronic retinal diseases usually require repetitive local dosing. Depending on factors such as dosing frequency, mode of administration, and associated costs, this can result in poor patient compliance. A better alternative involves using controlled-release drug delivery systems to reduce the frequency of intravitreal dosing and extend drug release. However, reaching the market stage is a time-consuming process. **Methods:** In this study, we employed two computational approaches to model and estimate the parameters governing the diffusion-controlled drug release from bi-layered microspheres. The case study involved microspheres composed of a chitosan core and a polycaprolactone (PCL) shell. The model drugs were bovine serum albumin and bevacizumab (an agent that slows neovascularization due to retinal disorders). Drug release from the microspheres is described by a mathematical model that was solved numerically using the finite difference and the finite element approaches. The parameter estimation was performed by nonlinear least-squares regression. **Results:** We used the estimated parameters to simulate the cumulative release under various conditions and optimize the device design to guide future experimental efforts and improve the duration of release beyond a target daily therapeutic release rate from the microspheres. **Conclusions:** We investigated the effects of polymeric layer sizes on drug release and provided recommendations for optimal sizes. We provide straightforward computational tools for others to reuse in designing bi-layered microspheres for intravitreal drug delivery needs in the treatment of chronic ocular neovascularization.

## 1. Introduction

Chronic diseases usually require repeated dosing to maintain therapeutic drug concentrations over extended periods and thus manage symptoms and prevent disease progression. This is particularly challenging in the treatment of chronic retinal disorders like age-related macular degeneration (AMD) and diabetic retinopathy, which involve neovascularization and require repetitive intravitreal injections. These frequent injections can cause fear and anxiety in patients and are associated with an increased risk of ocular complications [1]. Therefore, there is a critical need for drug delivery systems (DDSs) capable of maintaining therapeutic concentrations for extended periods, thereby improving patient compliance compared to classic formulations for administering drugs directly through systemic or localized routes.

Multiple polymer-based DDSs designed for intravitreal injection have been proposed in the literature to extend the release of anti-VEGF drugs targeting neovascularization [2]. These devices are generally made of biodegradable polymers like chitosan and polycaprolactone (PCL). Chitosan is a naturally derived polymer that is biocompatible and biodegradable [3] and has demonstrated versatility as a DDS across numerous biomedical applications [4,5,6,7,8]. PCL, on the other hand, is a hydrophobic, biocompatible polymer characterized by high permeability and a slow degradation profile [9] and has also been explored for multiple drug delivery applications [10,11,12,13,14]. PCL has a very slow hydrolytic degradation rate [15,16,17]. Bartnikowski et al. [17] combined data from multiple studies in the literature showing that less than 5% of PCL mass is lost after 10 months of in vitro degradation in cell-free media at body temperature; Lam et al. [18] showed that the 5% loss threshold is not passed until after nearly 2 years. Both chitosan [19,20,21,22,23] and PCL [24,25,26] have been explored as polymers for extending anti-VEGF drug release to prevent angiogenesis, as well as for the controlled release of anti-inflammatory agents [20,23,27,28] for other ocular diseases like uveitis [29]. Chitosan is positively charged, which provides a rationale for the extended release of negatively charged therapeutics, such as anti-VEGF agents, via electrostatic interactions [30]. However, only a few studies have combined these polymers into a multi-layered DDS (a core of one polymer coated successively by one or more layers of other polymers) to achieve extended drug release [30,31].

Achieving long-term, sustained delivery at therapeutic levels using DDSs requires consideration of the parameters that govern the drug release dynamics. In layered polymeric DDSs, the parameters include drug diffusion coefficients, the burst release percentage, and drug partition coefficients, which are not necessarily known beforehand; however, they influence the amount and rate of delivery at the target locations. Experimental testing of every possible design combination is impractical due to time and cost constraints. Mathematical modeling of drug release from DDSs enables predictive simulations of drug release behavior across a vast design space. These models can estimate the key parameters and optimize device design, reducing the amount of experimentation required while improving the treatment of chronic diseases.

Several mechanisms can govern drug release from polymeric DDSs, including diffusion, swelling, and erosion [2,32,33,34]. Here, we focus on diffusion-controlled release, where diffusion through the polymer(s) dominates the drug transport. Diffusion-controlled release is modeled by Fick’s second law, often assuming a constant diffusion coefficient in each material, perfect sink conditions in the medium, and negligible swelling and erosion of the DDS [33,34]. Analytical solutions exist for single-layered DDSs with ideal geometries [35,36,37,38,39,40] and multi-layered spherical DDSs under certain idealized conditions [41,42,43,44] and were reviewed by us [2]. Unfortunately, the analytical solutions are complicated to evaluate as they typically involve infinite sums of trigonometric functions. While these solutions rely on fixed values or assumed ratios among parameters, fewer studies have focused on parameter estimation from such single-layered [45,46,47] or multi-layered spheres [48,49].

This work addresses the challenge of improving the design of intravitreal DDSs through computational modeling, thereby reducing the design space for costly and time-consuming experiments. For evaluating cases that can be solved by analytical techniques, extending to other conditions without analytical solutions, and considering DDSs with more elaborate geometries, we turn to numerical approaches [50,51,52]. We use a finite difference approach in MATLAB for symmetric multi-layered spheres. We also use an alternative finite element approach in COMSOL to enable future extensions to layered geometries of other shapes and loading and boundary conditions that are not easily modeled in MATLAB. We present these two numerical approaches for solving the long-term drug release problem and estimating important parameters in diffusion-controlled core–shell spherical DDSs. As a case study, we apply these numerical methods to chitosan–PCL microspheres releasing bovine serum albumin (BSA) and bevacizumab [30], an anti-VEGF agent that slows neovascularization in cancer and eye disorders. The calculated parameters are estimated from published experimental data and used to explore the effects of the design variables—the sizes of the polymer layers—on the following drug release metrics: the cumulative release percentage and daily drug release rate. This framework supports the rational design of intravitreal DDSs to meet specific therapeutic thresholds, providing a generalizable toolset for both experimentalists and theoreticians working on sustained DDSs.

## 2. Methods

### 2.1. Geometry

**Figure 1:**
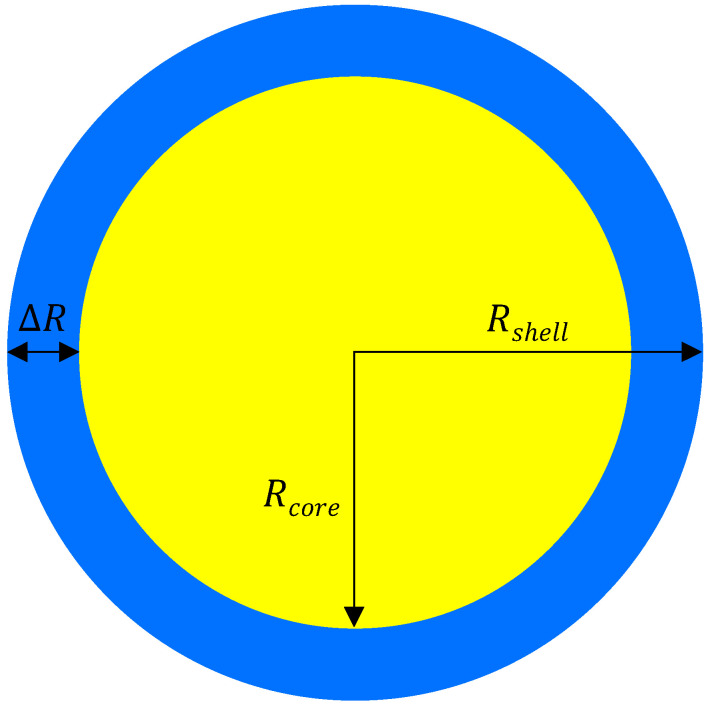
Two-dimensional cross-section of a core–shell microsphere. A drug is loaded only in the core. Yellow: core polymer and homogeneously distributed drug. Blue: shell polymer. *R*: radius. ΔR = $R_{shell}-R_{core}$. Note that the drug delivery system is not illustrated to scale.

The DDS used here is a core–shell microsphere (Figure 1). A single dose includes many of these microspheres, which are assumed to behave identically. The radius of the core is $R_{core}$, and that of the shell is $R_{shell}$. The shell thickness is denoted as $\Delta R=R_{shell}-R_{core}$. The minΔR=0; thus, $R_{shell}\geq R_{core}$. Note that ΔR=0 is just the single-layered microsphere case, where $R_{shell}=R_{core}$. Radial symmetry about the center of each microsphere is assumed.

As test cases for demonstrating our computational tools for designing DDSs for intravitreal controlled release, we used data from an experimental study performed by our team [30], in which the DDS consisted of a drug-loaded core made of chitosan and a PCL outer shell to protect the chitosan core from degradation and extend drug release. PCL hydrolytic degradation takes much longer than the 6-month time scale in the present study and in the study of Jiang et al. [30]; thus, PCL is assumed not to degrade. Chitosan has been shown to degrade by 6 months when not surrounded by a PCL outer shell, but it is protected when a shell is present [30]. Thus, we do not consider the case of ΔR=0 (a chitosan single-layered microsphere) as a test case for the diffusion-controlled model. Swelling is also assumed to be negligible for both polymers. Thus, diffusion-controlled release is the dominant release mechanism. In the study of Jiang et al. [30], the mean $R_{core}=5.10$ μm of chitosan, and the mean ΔR=1.25 μm of PCL. Two drugs were used in the study of Jiang et al. [30] and are considered here: BSA and bevacizumab.

### 2.2. Mathematical Model

Fick’s second law for time-dependent diffusion is as follows:

(1) $$\frac{\partial C}{\partial t}=D\nabla^{2}C$$

where $C(\vec{x},t)$ is the drug concentration, $\vec{x}$ refers to the spatial coordinates in either Cartesian or spherical coordinate systems, *t* is time, and *D* is a uniform diffusion coefficient. For two concentric layers of a symmetric sphere, this becomes

(2) $$\frac{\partial C}{\partial t}=\left\{\begin{array}{cc} \frac{D_{core}}{r^{2}}\frac{\partial }{\partial r}\left(r^{2}\frac{\partial C}{\partial r}\right), & 0\leq r\leq R_{core}\\ \frac{D_{shell}}{r^{2}}\frac{\partial }{\partial r}\left(r^{2}\frac{\partial C}{\partial r}\right), & R_{core}\leq r<R_{shell} \end{array}\right.$$

where $D_{core}$ and $D_{shell}$ are the diffusion coefficients for the core and shell layers, respectively, and *r* is the position along the radial dimension.

For the bi-layered or core–shell sphere, there are three boundary conditions to define for t>0 at r=0, $R_{core}$, and $R_{shell}$. At the center of the sphere (r=0), the radial symmetry boundary condition is applied:

(3) $$\frac{\partial C(0,t)}{\partial r}=0$$

The condition at the interface ($r=R_{core}$) between the two different polymeric domains is the flux continuity condition allowing for drug partitioning preferentially between the layers based on material properties:

(4) $$D_{core}\frac{\partial C_{core}\left(R_{core},t\right)}{\partial r}=D_{shell}\frac{\partial C_{shell}\left(R_{core},t\right)}{\partial r}$$

subject to

(5) $$C_{core}\left(R_{core},t\right)=\kappa C_{shell}\left(R_{core},t\right)$$

where $C_{core}\left(R_{core},t\right)$ is the drug concentration in the core at the interface, κ is the partition coefficient, and $C_{shell}\left(R_{core},t\right)$ is the drug concentration in the shell at the interface.

At the external surface of the sphere ($r=R_{shell})$, the perfect sink condition is assumed: $C(R_{shell},t)=0$. This means that all the drug released is immediately cleared away, which is a common assumption in biological tissues.

The initial concentration for the drug-loaded core and non-loaded shell is

(6) $$C\left(r,0\right)=\left\{\begin{array}{cc} C_{core,0}, & 0\leq r<R_{core}\\ 0, & R_{core}<r<R_{shell} \end{array}\right.$$

where $C_{core,0}$ is the initial concentration inside the core, which is set as $C_{core,0}=1$ arbitrary units (a.u.) for normalizing the concentration calculations. Note that this normalized value is the concentration that remains immediately after the burst release, which is assumed to be instantaneous at t=0.

Drug release is quantified in terms of cumulative release, as this metric is often reported in experimental studies. The cumulative release is calculated as follows:

(7) $$Q\left(t\right)=B+\left(100-B\right)\left(1-\frac{\;\int \int \int \;C(\vec{x},t)\;dV}{\;\int \int \int \;C(\vec{x},0)\;dV}\right)$$

where Q(t) is the cumulative percentage of drug released as a function of time, *B* is the burst release or the percentage of the total drug loaded that is released immediately upon in vivo administration or placement into an in vitro medium, *V* is the volume, and the last term refers to the fractional amount of drug remaining in the DDS at each time relative to that present in the DDS initially after the burst release. The total amount of drug in the core–shell microsphere at any time is calculated as the volume integral of the spatiotemporal distribution of $C(\vec{x},t)$ from the solution of Equations (2)–(6).

The amount of drug released over a time interval $A_{rel}\left(t,\Delta t\right)$ is determined using the cumulative release from each microsphere as follows:

(8) $$A_{rel}\left(t,\Delta t\right)=\frac{\left(Q\left(t\right)-Q\left(t-\Delta t\right)\right)A_{load,0}}{100},\;t>0$$

where Δt is the time interval and $A_{load,0}$ corresponds to the initial amount (in moles) of the drug loaded into a dose of multiple microspheres. We assumed that each microsphere has a uniform $C_{core,0}$ in its core. For diffusion and the cumulative release calculations, $C_{core,0}$ is dimensionless and is set to 1 to normalize by the dimensional initial concentration. Additionally, we assumed that the dimensional initial concentration in each microsphere core in units of moles per volume is the same across all microspheres, as this represents the loading capacity that is a material property. Microspheres of different core radii have different volumes over which the drug is distributed. Thus, we assumed that a sufficient number of microspheres comprise the dose to reach the $A_{load,0}$ amount. Different drugs have different molecular weights, and these were used when converting from initial amounts in mass units to mole units.

The release rate ${\dot{A}}_{rel}\left(t,\Delta t\right)$ can be expressed as follows:

(9) $${\dot{A}}_{rel}\left(t,\Delta t\right)=\frac{A_{rel}\left(t,\Delta t\right)}{\Delta t},\;t>0$$

which gives the release rate between two time points spaced by Δt or the approximation to the derivative of the cumulative release curve. The release rate is fastest at early times. As the Δt needed for burst release at time t=0 is assumed to be infinitesimally short, the instantaneous burst release rate is theoretically infinitely high (practically, it must be finite). Thus, Equation (9) is undefined at t=0, and we only considered the finite drug release rates calculated for t>0.

### 2.3. Numerical Methods

We solved the model for drug release from the core–shell microsphere DDS defined in Equations (2)–(6) numerically using two alternative approaches: finite differences in MATLAB 2024b and finite elements in COMSOL Multiphysics v6.2.

#### 2.3.1. Finite Difference Approach in MATLAB

For the radially symmetric core–shell sphere described in Section 2.1 with uniform diffusion coefficients in each layer and the initial and boundary conditions described in Section 2.2, the finite difference approach is relatively straightforward to implement in technical computing languages such as MATLAB or Python. Here, we used MATLAB. The first step in the approach is to normalize the spherical domain by scaling by $R_{shell}$ so that the domain is $0\leq r/R_{shell}\leq 1$. Then, a variable α is defined as

(10) $$\alpha =\left\{\begin{array}{cc} \alpha_{core}=\frac{D_{core}}{R_{shell}^{2}}, & 0\leq \frac{r}{R_{shell}}\leq \frac{R_{core}}{R_{shell}}\\ \alpha_{shell}=\frac{D_{shell}}{R_{shell}^{2}}, & \frac{R_{core}}{R_{shell}}\leq \frac{r}{R_{shell}}\leq 1 \end{array}\right.$$

to scale the diffusion coefficient by the outer radius and yield a spatially dimensionless form. Note that the time dimension is still included in α. As defined in Equation (10), α is a piecewise constant function.

In a technique called the method of lines [51,53], the partial differential equation (PDE) in Equation (2) with α substituted is transformed into a system of ordinary differential equations (ODEs) by discretizing the normalized spatial domain and using a finite differencing scheme to approximate the spatial derivatives at the discrete points in the domain. The resulting ODEs can be solved using any built-in MATLAB ODE solver; here, we used ode45. The classic, second-order-accurate central finite difference discretization scheme in spherical coordinates with uniform grid spacing Δr is as follows [39,50,54,55,56]:

(11) $$\frac{dC_{m}}{dt}=\left\{\begin{array}{cc} \frac{6\alpha_{0}}{\Delta r^{2}}\left(C_{1}-C_{0}\right), & m=0\\ \frac{\alpha_{0}}{m\Delta r^{2}}\left(\left(m+1\right)C_{m+1}-2mC_{m}+\left(m-1\right)C_{m-1}\right),\; & m=1,\;2,\;\ldots ,M-1\\ 0, & m=M \end{array}\right.$$

where $C_{m}\left(t\right)$ is the numerical approximation to $C(r_{m},t)$ at the grid point $r_{m}=m\Delta r$ for m=0, 1, ..., *M* inside the normalized and discretized spherical domain with continuous time *t*, *M* is the number of discretizations in the domain, and Δr=1/M. Equation (11) holds for a constant value $\alpha_{0}=D/R_{shell}^{2}$ that is uniform throughout a spherical domain. Ford Versypt and Braatz [56] generalized Equation (11) to allow for any function α(r,t), including a piecewise constant function like Equation (10). However, this technique assumes that the grid spacing Δr does not change. A fixed value of Δr could be implemented for select values of $R_{core}$ and $R_{shell}$; however, here it was desirable to make the algorithm work for arbitrary combinations of $R_{core}$ and $R_{shell}$ to enable exploration of the design space of these sizes. Additionally, the interface boundary condition in Equations (4) and (5) needed to be satisfied. Thus, we adapted Equation (11) as the following schemes in the two layers for solving Equations (2)–(5):

(12) $$\frac{dC_{i}}{dt}=\left\{\begin{array}{cc} \frac{6\alpha_{core}}{\Delta r_{core}^{2}}\left(C_{1}-C_{0}\right), & i=0\\ \frac{\alpha_{core}}{i\Delta r_{core}^{2}}\left(\left(i+1\right)C_{i+1}-2iC_{i}+\left(i-1\right)C_{i-1}\right),\; & i=1,\;2,\;\ldots ,I_{\mathrm{core}}-1\\ \kappa \frac{dC_{j}}{dt}{|}_{j=0}, & i=I_{core} \end{array}\right.$$

(13) $$\frac{dC_{j}}{dt}=\left\{\begin{array}{cc} \frac{2\Gamma C_{j+1}-2\left(\Gamma +\kappa \right)C_{j}+2C_{i-1}}{\kappa \frac{\Delta r_{core}^{2}}{\alpha_{core}}+\Gamma \frac{\Delta r_{shell}^{2}}{\alpha_{shell}}}, & j=0\;\mathrm{and}\;i=I_{core}\\ \frac{\alpha_{shell}}{r_{j}\Delta r_{shell}}\left(C_{j+1}-C_{j-1}\right)+\frac{\alpha_{shell}}{\Delta r_{shell}^{2}}\left(C_{j+1}-2C_{j}+C_{j-1}\right),\; & j=1,\;2,\;\ldots ,J_{\mathrm{shell}}-1\\ 0, & j=J_{shell} \end{array}\right.$$

where $C_{i}\left(t\right)$ is the numerical approximation to $C(r_{i},t)$ at the grid points $r_{i}=i\Delta r_{core}$ for i=0, 1, ..., $I_{core}$ inside the core for $0\leq r/R_{shell}\leq R_{core}/R_{shell}$, $\Delta r_{core}=1/I_{core}$ is the normalized grid spacing in the core, $I_{core}$ is the number of spatial discretizations in the core, $C_{j}\left(t\right)$ is the numerical approximation to $C(r_{j},t)$ at the grid points $r_{j}=I_{core}\Delta r_{core}+j\Delta r_{shell}$ for j=0, 1, ..., $J_{shell}$ inside the shell for $R_{core}/R_{shell}\leq r/R_{shell}<R_{shell}/R_{shell}$, $\Delta r_{shell}=1/J_{shell}$ is the normalized grid spacing in the shell, $J_{shell}$ is the number of spatial discretizations in the shell, and

(14) $$\Gamma =\left(\frac{I_{core}\Delta r_{core}+\Delta r_{core}}{I_{core}\Delta r_{core}-\Delta r_{shell}}\right)\left(\frac{\alpha_{shell}\Delta r_{core}}{\alpha_{core}\Delta r_{shell}}\right)$$

The schemes for the interior points in the shell layer (j=1, 2, ..., $J_{shell}-1$) and at the interface ($i=I_{core}$ and j=0) and the selection of grid spacing and relationships between the grid spacing in the two layers to maintain second-order accuracy are derived in Section S1 in the Supplementary Materials using techniques from [39,57,58,59]. Figure S1 in the Supplementary Materials shows a schematic representation of the core and shell interior points, the interface location, and the application of the fictitious node method at the interface.

In addition to the initial concentrations from Equation (6) for the core and shell, the MATLAB implementation requires that the initial concentration at the interface be explicitly defined. The initial values at the interface are derived in Section S1 in the Supplementary Materials as the weighted averages of the initial values in the core and the shell, with the weighting dependent on the partition coefficient κ and the transport properties in the layers (Equations (S26) and (S27) in the Supplementary Materials):

(15) $$C\left(r,0\right)=\left\{\begin{array}{cc} \frac{\kappa C_{core,0}}{\kappa +\gamma }, & r=R_{core}\;\mathrm{in}\;\mathrm{core}\\ \frac{C_{core,0}}{\kappa +\gamma }, & r=R_{core}\;\mathrm{in}\;\mathrm{shell} \end{array}\right.$$

where γ is a ratio of the diffusion coefficients and the grid spacing in the two layers, defined in Equation (S18) in the Supplementary Materials.

Numerical techniques were used to evaluate the volume integrals in Equation (7) with discrete values of $C(\vec{x},t)$ from the MATLAB finite difference solution. The MATLAB function simps [60] approximates each volume integral using Simpson’s method for numerical integration. In spherical coordinates, the volume integral becomes

(16) $$\begin{array}{cc} \;\int \int \int \;C(\vec{x},t)\;dV & =\int_{0}^{R_{shell}}\int_{0}^{2\pi }\int_{0}^{\pi }C\left(r,t\right)r^{2}\sin\theta \;d\theta d\phi dr \end{array}$$

(17) $$\begin{array}{cc} \; & =\frac{4\pi }{3}\left(\int_{0}^{R_{core}}C\left(r,t\right)r^{2}\;dr+\int_{R_{core}}^{R_{shell}}C\left(r,t\right)r^{2}\;dr\right) \end{array}$$

The final term allows for different grid spacing in the two layers and for non-uniform initial values of C(r,0) from Equation (6).

#### 2.3.2. Finite Element Approach in COMSOL Multiphysics

COMSOL Multiphysics was used to solve the model equations using the finite element method with P1 elements. A P1 element uses a linear polynomial to approximate the solution within its boundaries [61]. The model geometry is a two-dimensional (2D) axisymmetric structure, which reduces the three-dimensional (3D) problem to a 2D problem by leveraging the symmetry about the vertical axis in the 2D plane. A 2D representation was selected instead of a one-dimensional version as we wanted to generalize to other 2D axisymmetric (and 3D) geometries in the future. The solution procedure involves discretizing the spatial domain into simple geometric elements that are interconnected at common points with two or more elements. This process results in a system of algebraic equations that is solved simultaneously. A triangular mesh is used to define the 2D elements in the computational domain. In our implementation, the maximum element size allowed was 0.1 mm, while the minimum element size allowed was $2.4\times 10^{-5}$ mm based on the work of Van Kampen et al. [62], who also used COMSOL for modeling sustained intravitreal delivery from a DDS. The COMSOL “Transport of Diluted Species” interface was used as the physics package in the software to set up the PDE in Equation (2), specify the boundary and initial conditions described in Section 2.2, and solve for the time-dependent drug concentration values in each layer of the DDS. At the interface between the layers, the boundary condition option PartitionCondition was used to specify κ.

Numerical techniques were used to evaluate the volume integrals in Equation (7) with discrete values of $C(\vec{x},t)$ from the COMSOL finite element solution. The COMSOL built-in option Non-local coupling: integration was used to evaluate the volume integral with the spatially varying concentration profile of $C(\vec{x},t)$ over the 2D axisymmetric domain that represents a microsphere.

The finite element approach in COMSOL Multiphysics and the finite difference approach in MATLAB were compared under the same parameter conditions. The drug concentration results at different locations inside the microspheres were used for verification purposes and were in good agreement (Section S2 and Figure S2 in the Supplementary Materials). For additional verification, the cumulative drug release profiles from the numerical methods implemented in MATLAB and COMSOL were compared to an analytical solution [41]. All three approaches yielded overlapping results for two arbitrary sets of parameters, demonstrating strong concordance across methodologies (Section S2 and Figure S3 in the Supplementary Materials).

### 2.4. Sensitivity Analysis

We used one-at-a-time sensitivity approaches in MATLAB and COMSOL and the global variance-based Sobol’s method [63,64,65] implemented in COMSOL to assess the sensitivity of the model output to the input parameters. A sensitivity matrix *S* is defined with the general form:

(18) $$S=\left[\begin{array}{ccc} \frac{\partial f(x_{1},p)}{\partial p_{1}} & \ldots & \frac{\partial f(x_{1},p)}{\partial p_{m}}\\ \vdots & \ddots & \vdots \\ \frac{\partial f(x_{n},p)}{\partial p_{1}} & \ldots & \frac{\partial f(x_{n},p)}{\partial p_{m}} \end{array}\right]$$

where $f(x_{i},p)$ is a model output evaluated at input variable value $x_{i}$ for i=1, 2, ..., *n* subject to the set of parameters $p=p_{1},p_{2},\ldots ,p_{m}$. Generally, the input variables include the independent variable time *t*, the selected drug, and the geometric properties of the DDS: $R_{core}$ and $R_{shell}$. Here, we fixed $R_{core}$ and $R_{shell}$ to be the properties of the bi-layered microspheres from Jiang et al. [30] described in Section 2.1. For the model output of interest, we considered the cumulative release at the single time point of 28 days, Q(28), from Equation (7) and the model solution of the concentration distribution from Equations (1)–(6) and the set of parameters $p=\{B,D_{core}=D_{Chi},D_{shell}=D_{PCL},\kappa \}$. The sensitivity analysis was performed for three regimes of diffusion coefficients in the two layers: $D_{core}<<D_{shell}$, $D_{core}\approx D_{shell}$, and $D_{core}>>D_{shell}$.

For the MATLAB local sensitivity analysis, finite differences were used to approximate the derivatives in Equation (18), and each parameter was increased one at a time by 1%. Thus, the normalized local sensitivity $N_{Q(28,p)}$ for the single output for each drug and four parameters is as follows:

(19) $$N_{Q(28,p)}=\left[\begin{array}{c} \frac{Q\left(28,1.01\times B,D_{Chi},D_{PCL},\kappa \right)-Q\left(28,p\right)}{0.01\times Q(28,p)}\\ \frac{Q\left(28,B,1.01\times D_{Chi},D_{PCL},\kappa \right)-Q\left(28,p\right)}{0.01\times Q(28,p)}\\ \frac{Q\left(28,B,D_{Chi},1.01\times D_{PCL},\kappa \right)-Q\left(28,p\right)}{0.01\times Q(28,p)}\\ \frac{Q\left(28,B,D_{Chi},D_{PCL},1.01\times \kappa \right)-Q\left(28,p\right)}{0.01\times Q(28,p)} \end{array}\right]$$

In COMSOL, the Morris one-at-a-time (MOAT) method [66,67,68] and Sobol’s method were both used from the uncertainty quantification module [69]; we allowed each parameter to be described by a normal distribution and a 1% standard deviation around the baseline value. The cumulative distribution function bounds were defined to constrain the parameter range within ±1% of the baseline value, with the lower bound set at 99% and the upper bound set at 101% of the baseline.

### 2.5. Parameter Estimation

For the estimation of the parameters p={burst release *B*, drug diffusion coefficients in the core $D_{core}$ and the shell $D_{shell}$, and the partition coefficient κ}, we used an ordinary least-squares objective function with the following general form:

(20) $$\min_{p}\sum_{i}{\left[f\left(xdata_{i},p\right)-ydata_{i}\right]}^{2}$$

where $f(xdata_{i},p)$ is the model output evaluated at the values of input variables at which experimental measurements $ydata_{i}$ are collected and *i* denotes discrete data points. Here, the data were from Jiang et al. [30], and the model outputs of interest were the cumulative release (Equation (7)) values at times $xdata_{i}$ when the measurements for experimental cumulative drug release $ydata_{i}$ were obtained. As in the sensitivity analysis, $R_{core}$ and $R_{shell}$ were fixed, and the parameter estimation process was conducted for both BSA and bevacizumab. Equation (20) minimizes the sum of squared residuals, which we also refer to as the “error”.

The MATLAB built-in function lsqcurvefit was used for estimating the parameters that minimized the least-squares error (Equation (20)). The parameter values were normalized by dividing them by scaling factors specific to each parameter so that the maximum of the parameter range was set to 1. The estimated values were then multiplied by the scaling factors to yield the optimized parameter values. Tolerance was set to 0.001, and the maximum number of objective function evaluations for each parameter update was set to 800. All other optimization settings were at their default values.

We performed a preliminary parameter estimation, described in Section S3 in the Supplementary Materials, over a broad parameter space that yielded feasible cumulative release profiles (Figure S4 and Table S1 in the Supplementary Materials). The results from the preliminary parameter estimation (Figures S5 and S6 and Table S2 in the Supplementary Materials) allowed us to reduce the parameter space of the problem before refining the parameter estimates. We performed a subsequent parameter estimation using a multi-start process with 50 initial guesses determined by Latin hypercube sampling of the parameter values bounded within the new limits in Table 1 with log uniform sampling across each parameter. The parameters with the lowest sensitivity were fixed at κ=1 and $D_{PCL}=1000\times D_{Chi}$ leaving $p=\{B,D_{core}=D_{Chi}\}$ as the parameters to be estimated in the refined parameter estimation. Upon completing the 50 multi-start parameter estimations, we compared the least-squares error values for the parameter sets to which the lsqcurvefit algorithm converged, subject to each of the 50 initial parameter guesses. The parameter sets from the multi-start parameter estimations that yielded error values within 5% of the minimum error were deemed acceptable. The resulting parameter values from the acceptable sets were averaged and then simulated for the average model results. Additionally, the parameters from the simulation with the lowest error value were considered the best model parameters. After comparing the best model and the average model, the average parameters were used in all further model simulations.

In COMSOL, we used the built-in global least-squares objective function in the optimization module to perform the parameter estimation (Equation (20)) for the four model parameters $p=\{B,D_{core}=D_{Chi},D_{shell}=D_{PCL},\kappa \}$ in the preliminary parameter estimation and the two sensitive parameters $p=\{B,D_{core}=D_{Chi}\}$ for the refined multi-state parameter estimation procedure. The parameter values were normalized by dividing them by scaling factors specific to each parameter; in COMSOL, these scaling factors were chosen such that the midpoint of the parameter range was set to 1. Tolerance was set to 0.001, and the maximum number of function evaluations was set to 1000.

### 2.6. Parameter Uncertainty Quantification

To quantify the uncertainty in the parameters estimated following the methods of Section 2.5, we again used the sensitivity matrix *S* from Equation (18). Here, the *S* was approximated using the finite difference approach in MATLAB with 1% perturbation in each of the parameters to numerically determine the Jacobian, and the model outputs of interest *f* included the cumulative release Q(t) values from Equation (7) at multiple time points. The input variables $x_{i}$ are discrete time points that correspond to 11 measurement times for cumulative release data from the study of Jiang et al. [30]. The covariance of the cumulative release experimental data is collected in a matrix ψ. With *S* and ψ, the variance–covariance matrix cov(p) for parameters *p* is approximated as follows [70]:

(21) $$\mathrm{cov}\left(p\right)={\left(S^{T}S\right)}^{-1}S^{T}\psi S{\left(S^{T}S\right)}^{-1}$$

where *T* denotes the matrix transpose operation. The diagonal elements of this square matrix cov(p) give the variance of the parameters and were used to determine the parameters’ standard deviation. Finally, using this standard deviation in conjunction with the Student’s t inverse cumulative distribution function, the 95% confidence interval for each estimated parameter was calculated.

## 3. Results

### 3.1. Sensitivity Analysis

**Figure 2:**
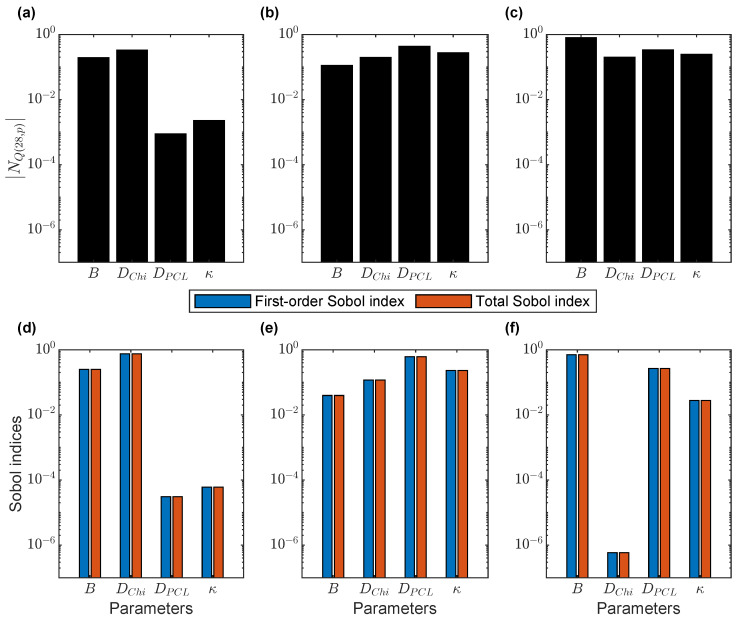
Sensitivity analysis by local and global methods for three regimes of relative magnitudes of diffusion coefficients in the chitosan (Chi) core and the polycaprolactone (PCL) shell. Top row: Absolute value of the normalized sensitivity of the cumulative drug release at 28 days $|N_{Q(28,p)}|$ obtained from the finite difference solution in MATLAB. Each parameter was varied one at a time using a 1% increase. Bottom row: Sobol indices for the sensitivity analysis of parameters affecting cumulative drug release at 28 days obtained using the finite element solution in COMSOL. For each case, B=10 and κ=1. First column: $D_{Chi}<<D_{PCL}$. Second column: $D_{Chi}=D_{PCL}$. Third column: $D_{Chi}>>D_{PCL}$. (**a**,**d**) $D_{Chi}=1\times 10^{-15}$ cm^2^/s and $D_{PCL}=1000\times D_{Chi}=1\times 10^{-12}$ cm^2^/s, (**b**,**e**) $D_{Chi}=1\times 10^{-14}$ cm^2^/s and $D_{PCL}=D_{Chi}$, and (**c**,**f**) $D_{Chi}=1\times 10^{-12}$ cm^2^/s and $D_{PCL}=0.001\times D_{Chi}=1\times 10^{-15}$ cm^2^/s. *B*: burst release. κ: partition coefficient. $D_{Chi}=D_{core}$: drug diffusion coefficient in the chitosan core. $D_{PCL}=D_{shell}$: drug diffusion coefficient in the PCL shell.

We observed that the normalized sensitivity of the cumulative drug release at 28 days (Equation (19)) depended on the relative magnitudes of the diffusion coefficients in the chitosan core and the PCL shell (Figure 2). We identified three regimes for these relative magnitudes: $D_{core}<<D_{shell}$, $D_{core}\approx D_{shell}$, and $D_{core}>>D_{shell}$ (Section S4 and Figure S7 in the Supplementary Materials). For all the cases shown, the burst and the partition coefficient were tested at the same baseline values of 10 and 1, respectively. The MATLAB local sensitivity results are shown in Figure 2a–c, and the Sobol indices for the global sensitivity results from COMSOL are shown in Figure 2d–f. The sensitivity values from both approaches followed the same patterns regarding the ordering of the sensitivities of the four parameters; however, the insensitive parameters from the Sobol analysis had smaller magnitudes for the sensitivity indices than the corresponding values from the MATLAB local sensitivity analysis. Furthermore, the agreement between first-order and total Sobol indices across all three regimes suggests negligible interaction effects between parameters, indicating that each had an independent influence on cumulative release. These results are consistent with the results from the local MOAT analysis in COMSOL (Section S5 and Figure S8 in the Supplementary Materials).

The most influential parameter depended on the specific regime considered. When drug release was limited by drug diffusion in the chitosan core ($D_{Chi}<<D_{PCL}$, Figure 2a,d), the drug diffusion coefficient in the chitosan core $D_{Chi}$ was the most influential parameter, with burst release *B* having a comparable effect. In contrast, the drug diffusion in the PCL shell $D_{PCL}$ and the partition coefficient κ had negligible impacts on the cumulative drug release at 28 days. When the diffusion coefficients were the same ($D_{Chi}=D_{PCL}$, Figure 2b,e), the sensitivity shifted, and the drug diffusion coefficient in the PCL shell $D_{PCL}$ became the dominant parameter, followed by the partition coefficient κ and the drug diffusion coefficient in chitosan $D_{Chi}$. In this case, the burst release *B* had the lowest impact on the cumulative drug release at 28 days. When drug release was limited by drug diffusion in the PCL shell ($D_{Chi}>>D_{PCL}$, Figure 2c,f), the burst release *B* was the most sensitive parameter, followed by the drug diffusion coefficient in the PCL shell $D_{PCL}$ and partition coefficient κ. In this regime, the drug diffusion coefficient in the chitosan core $D_{Chi}$ had the smallest effect on the cumulative drug release at 28 days.

### 3.2. Parameter Estimation

The preliminary parameter estimation considering all four parameters for both BSA and bevacizumab (Section S3 in the Supplementary Materials) shows that $D_{Chi}<<D_{PCL}$ best fits the chitosan–PCL core–shell microsphere DDS data used here (Figures S5 and S6 and Table S2 in the Supplementary Materials). This parameter regime was insensitive to the parameters $D_{PCL}$ and κ (Figure 2a,d), meaning that their values did not substantially influence the model. Therefore, we set κ=1 and $D_{PCL}=1000\times D_{Chi}$ and only further refined the parameter estimates for $D_{Chi}$ and *B*. This reduced model complexity and accelerated the parameter estimation and solution procedure without compromising accuracy.

**Figure 3:**
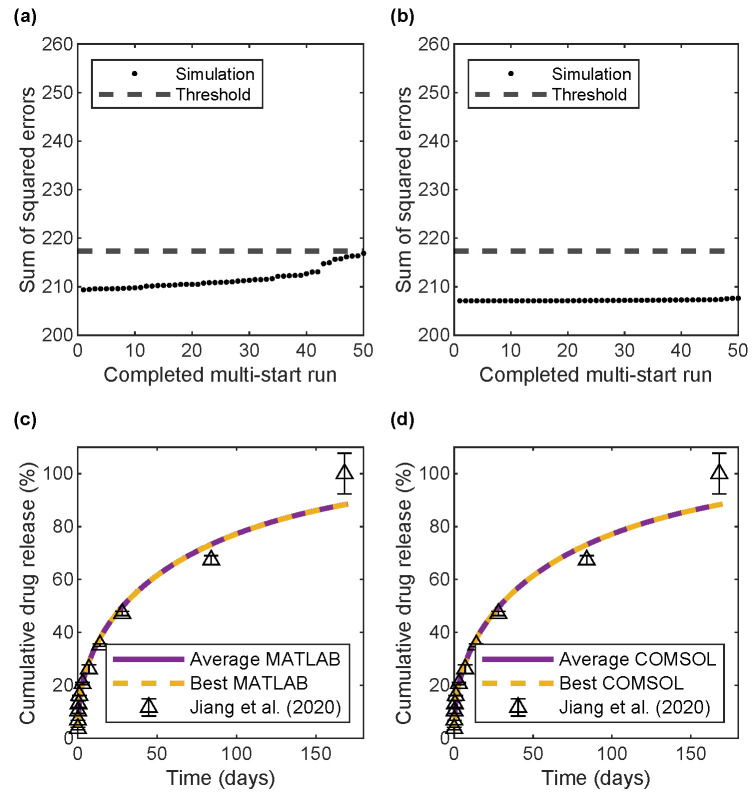
Error values and cumulative drug release profiles for BSA release after 50 multi-start parameter estimations. (**a**) Error values in MATLAB. (**b**) Error values in COMSOL. (**c**) Average and best MATLAB models compared to experimental data. (**d**) Average and best COMSOL models compared to experimental data. Experimental data from Jiang et al. [30].

**Figure 4:**
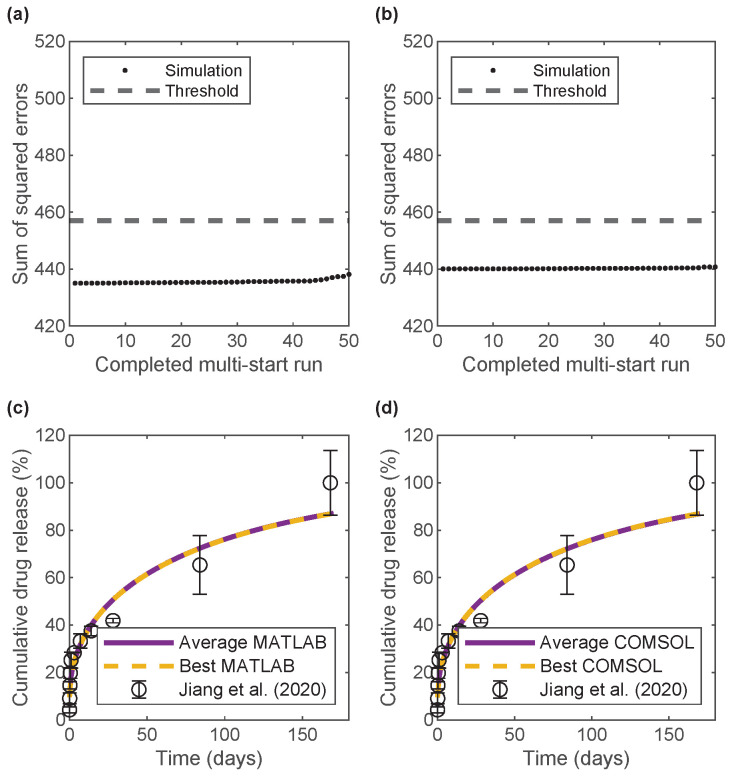
Error values and cumulative drug release profiles for bevacizumab release after 50 multi-start parameter estimations. (**a**) Error values in MATLAB. (**b**) Error values in COMSOL. (**c**) Average and best MATLAB models compared to experimental data. (**d**) Average and best COMSOL models compared to experimental data. Experimental data from Jiang et al. [30].

All the error values after each completed parameter estimation for BSA release in MATLAB and COMSOL (Figure 3a,b, respectively) and for bevacizumab release (Figure 4a,b) were within a 5% error threshold and, therefore, were used when averaging the parameters. Thus, the parameter estimation process was insensitive to variations in initial guesses for this regime of diffusion coefficients. For both MATLAB and COMSOL, the average and best (minimum error over all the multi-start parameter estimations) model predictions overlapped and were close to the experimental data (Figure 3c,d and Figure 4c,d), indicating that the implementation of the model in both software programs predicted the same BSA and bevacizumab release from the bi-layered microspheres, in agreement with the data. Furthermore, Table 2 shows the similarity between the average and best model parameters for each software program and the similarity between the best models from both software programs.

Table 2 also shows the 95% confidence intervals for the uncertainty in parameter values, as calculated with the MATLAB results according to Section 2.6. For both BSA and bevacizumab, burst release had a relatively narrower confidence interval than that for the drug diffusion coefficient in the chitosan core. For both drugs, the confidence intervals did not cross into the negative regions for any parameter. Among the parameters estimated in COMSOL, none of them fell outside the 95% confidence intervals obtained from MATLAB.

### 3.3. Predictive Capabilities

The computational model was used to explore the impact of the sizes of both the chitosan and PCL layers on the cumulative drug release and the drug release rate to optimize polymer layer sizes for bevacizumab release. The predicted bevacizumab release rate was obtained from MATLAB using Equations (8) and (9). Alternatively, the release rate could be computed in COMSOL as the line integral of the total normal flux multiplied by the molecular weight of bevacizumab. When simulating microspheres of different sizes in COMSOL, the computed flux must also be scaled by a geometric factor that accounts for changes in surface area due to variations in sphere volume relative to the baseline size. The bevacizumab loaded amount $A_{load,0}$ for all simulations was 1.03 mg. This loading amount is feasible and is representative of the range of 0.99–1.61 mg of BSA or bevacizumab loaded into 4 mg of chitosan or chitosan–PCL microspheres by Jiang et al. [30].

The target for the optimal design was to maintain the drug release rate above the estimated threshold for as long as possible while ensuring that a substantial portion of the total drug was delivered based on cumulative release. The threshold for drug release was derived from a previously published study that established that an injection of 12.5 μg of bevacizumab was sufficient to reduce neovascularization leakage during one week in patients with proliferative diabetic retinopathy [71]. By dividing the injected amount over 7 days, the approximate threshold can be rounded to 2.0 μg/day. This is consistent with the findings of Carichino et al. [72], who calculated that a retinal anti-VEGF production rate of 1.12–2.62 μg/day would be as effective as an injection of ranibizumab (another therapeutic that slows neovascularization). Here, the cumulative drug release threshold was set at 90%, representative of practical drug release scenarios, and this threshold was also used in another study of sustained intravitreal protein delivery from a DDS [62].

**Figure 5:**
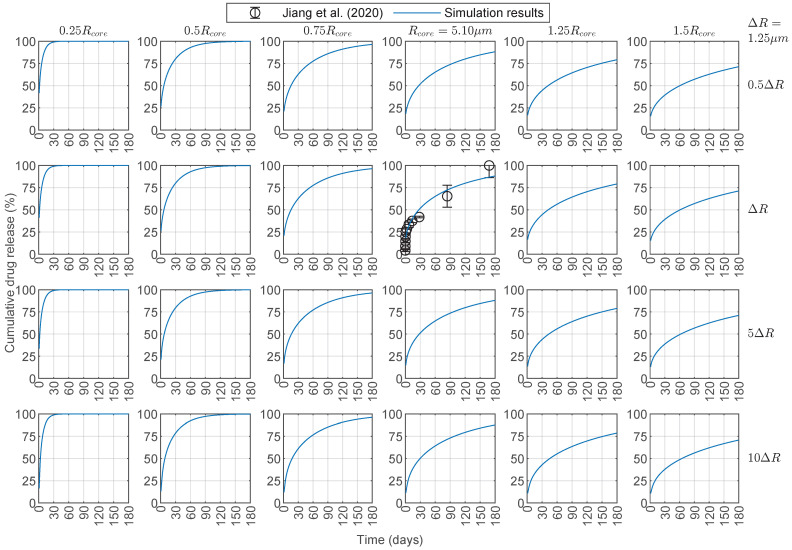
Cumulative drug release profiles for bevacizumab released from core–shell microspheres with drug loaded in the core only. Results were obtained in MATLAB with $D_{Chi}=2.6\times 10^{-15}$ cm^2^/s, $D_{PCL}=2.6\times 10^{-12}$ cm^2^/s, B=10%, and κ=1 (average model parameters from Table 2). Experimental data for $R_{core}=5.10$ μm and ΔR = $R_{shell}-R_{core}=1.25$ μm are from Jiang et al. [30]. Each panel shows profiles for different chitosan–PCL configurations where the core radius and shell thickness are varied by multipliers applied to the baseline dimensions $R_{core}$ and ΔR from Jiang et al. [30]. The core radii are labeled on the columns, and the shell thicknesses are labeled on the rows. $D_{Chi}=D_{core}$: drug diffusion coefficient in the chitosan (Chi) core. $D_{PCL}=D_{shell}$: drug diffusion coefficient in the polycaprolactone (PCL) shell. *B*: burst release. κ: partition coefficient.

The results in this section were simulated in MATLAB using the average model parameters for bevacizumab in Table 2. Increasing the radius of the chitosan core (left to right in Figure 5) resulted in slower cumulative drug release due to the greater distance the drug had to diffuse through the polymer. A similar but weaker effect was observed when increasing the PCL shell layer (top to bottom in Figure 5). A large increase in the PCL shell would be required to slow the cumulative drug release substantially, consistent with the model’s insensitivity to $D_{PCL}$ in the parameter regime where the chitosan core controls the release ($D_{core}<<D_{shell}$). For the fastest cumulative drug release (top left in Figure 5), the 90% cumulative release threshold was achieved between 12 and 13 days, whereas, for the slowest cumulative drug release (bottom right in Figure 5), only about 70% of the loaded drug was released after 6 months. We extended the simulation time to 360 days (Figure S9 in the Supplementary Materials), and the slowest case increased to 85% cumulative release by that time.

**Figure 6:**
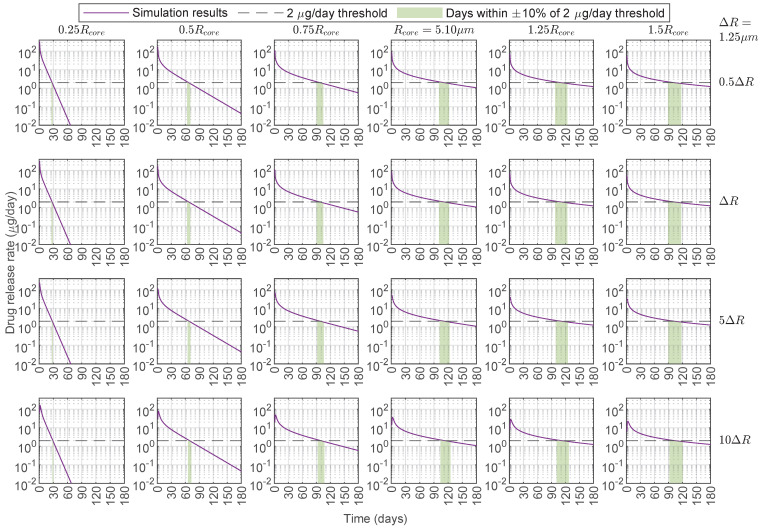
Drug release rate profiles for bevacizumab released from core–shell microspheres with drug loaded in the core only. Results were obtained in MATLAB with $D_{Chi}=2.6\times 10^{-15}$ cm^2^/s, $D_{PCL}=2.6\times 10^{-12}$ cm^2^/s, B=10%, and κ=1 (average model parameters from Table 2). Each panel shows profiles for different chitosan–PCL configurations where the core radius and shell thickness are varied by multipliers applied to the baseline dimensions $R_{core}=5.10$ μm and ΔR = $R_{shell}-R_{core}=1.25$ μm from Jiang et al. [30]. The core radii are labeled on the columns, and the shell thicknesses are labeled on the rows. In each panel, the purple curve is the simulation results, the black dashed line shows the 2 μg/day drug release rate threshold, and the shaded green region highlights the days within ±10% of the 2 μg/day drug release rate threshold. $D_{Chi}=D_{core}$: drug diffusion coefficient in the chitosan (Chi) core. $D_{PCL}=D_{shell}$: drug diffusion coefficient in the polycaprolactone (PCL) shell. *B*: burst release. κ: partition coefficient.

Because of the assumption that $C_{core,0}$ is the same for all microspheres, those with a larger $R_{core}$ were loaded with more drug, and fewer of these microspheres were considered in a dose to maintain the same $A_{load,0}$ for all simulations. Reducing the chitosan core radius below the baseline resulted in a faster release, shortening the duration of the therapeutic delivery rate (Figure 6). In contrast, increasing the chitosan radius beyond 1.5 times its baseline value was not substantially beneficial for the drug release rate. Furthermore, variations in the PCL layer had little effect on the drug release rate within the parameter regime that best fits the data, and a substantial increase in the PCL layer would be required to observe any appreciable change in the release rate. For instance, for baseline chitosan and PCL, a 10-fold increase of the PCL layer thickness only extended the time above the target 2 μg/day drug release rate from 111 days to 114 days. This limited PCL layer dependence was not surprising given that no drug was loaded into the PCL layer and $D_{core}<<D_{shell}$. Additionally, considering a ±10% window around the release rate threshold gave a range of days at which the window of the minimum therapeutic release threshold was reached, and the duration of this therapeutic target range increased with the chitosan radius (green shaded region in Figure 6). Finally, all microspheres with a chitosan radius at the baseline value or larger maintained their drug release rate over 1 μg/day by the end of the simulations at 180 days (Figure 6). We extended the simulation time to 360 days and lowered the drug release rate threshold to 1 μg/day (Figure S10 in the Supplementary Material), and all the chitosan–PCL configurations considered reached that threshold by 240 days.

**Figure 7:**
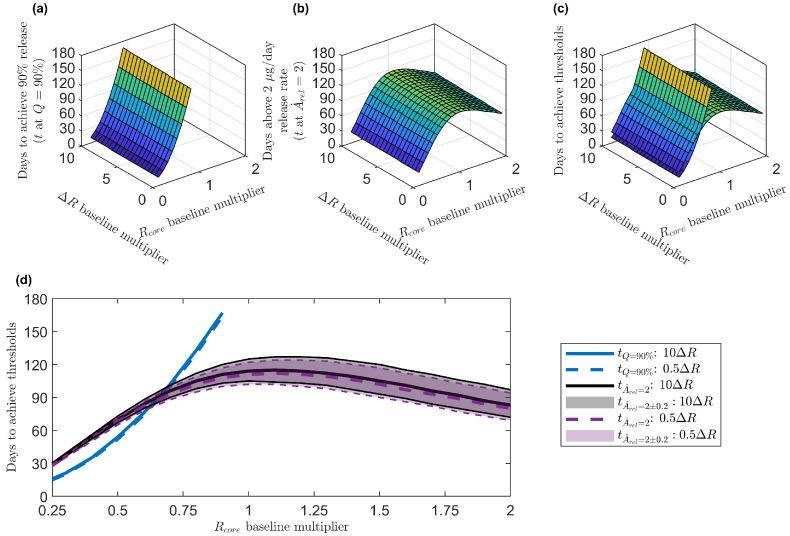
Time in days to reach cumulative release (*Q*) and release rate (${\dot{A}}_{rel}$) thresholds for different chitosan–PCL configurations (including those from Figure 5 and Figure 6) where the core radius and shell thickness are varied by multipliers applied to the baseline dimensions $R_{core}=5.10$ μm and ΔR = $R_{shell}-R_{core}=1.25$ μm from Jiang et al. [30]. (**a**) Time in days to reach cumulative release threshold of Q=90% as a function of $R_{core}$ and ΔR baseline multipliers. (**b**) Time in days to reach release rate threshold of ${\dot{A}}_{rel}=2$ μg/day as a function of $R_{core}$ and ΔR baseline multipliers. (**c**) Three-dimensional view of the surfaces from panels (**a**,**b**) combined. (**d**) Two-dimensional view of panel (**c**) in the projection onto the time vs. $R_{core}$ baseline multiplier axes. ΔR baseline multipliers of 0.5 and 10 are shown in panel (**d**) along with shaded regions denoting the intervals where the release rates are within ±10% of the threshold. The blue solid line and the blue dashed line are the simulation results for the 90% cumulative release threshold for 10×ΔR and 0.5×ΔR, respectively. The black solid line and the purple dashed line are the simulation results for the 2 μg/day release rate threshold for 10×ΔR and 0.5×ΔR, respectively. The gray shaded region and the purple shaded region highlight the days within ±10% of the 2 μg/day drug release rate threshold for 10×ΔR and 0.5×ΔR, respectively. Results for all panels were obtained in MATLAB with $D_{Chi}=2.6\times 10^{-15}$ cm^2^/s, $D_{PCL}=2.6\times 10^{-12}$ cm^2^/s, B=10%, and κ=1 (average model parameters from Table 2). $D_{Chi}=D_{core}$: drug diffusion coefficient in the chitosan (Chi) core. $D_{PCL}=D_{shell}$: drug diffusion coefficient in the polycaprolactone (PCL) shell. *B*: burst release. κ: partition coefficient.

The time to reach the 90% cumulative release threshold increased monotonically with $R_{core}$ and ΔR, with a stronger effect of $R_{core}$ (Figure 7a). The duration of time above the 2 μg/day release rate threshold reached a maximum at around 1.2 times the baseline chitosan radius (Figure 7b); variations in the PCL layer thickness had little effect. Figure 7c shows the surface plots for the threshold cumulative drug release and drug release rate from Figure 7a,b to visualize their intersection. Figure 7d shows two of the three axes of Figure 7c with the largest and smallest ΔR baseline multipliers plotted. The shading around the curves indicates the intervals where the release rate is within ±10% of the target 2 μg/day. While the surface plot in Figure 7b goes through a maximum, Figure 7d shows that values of ≈1–$1.4\times R_{core}$ have similar numbers of days above the drug release rate threshold, particularly when considering the intervals around the target threshold.

One possible optimal design is the chitosan–PCL configuration that yielded the intersection of the surfaces in Figure 7c and the curves in Figure 7d, i.e., the times to reach the cumulative drug release and drug release rate thresholds were the same. This occurred for a chitosan–PCL bi-layered microsphere configuration with $\approx 0.65\times R_{core}$ and 1×ΔR (as there was not substantial dependence on the PCL layer thickness). This configuration sustained a drug release rate over 2 μg/day and achieved 90% drug depletion within 90 days, minimizing the time between falling below the therapeutic release rate and fully depleting the microspheres of their drug payloads. An alternative definition of an “optimal” chitosan–PCL configuration is where the time above the drug release rate threshold reaches a maximum, as long as the microspheres have not surpassed the 90% cumulative release threshold. For the release rate threshold of 2 μg/day for more than 100 days, this optimal chitosan–PCL bi-layered microsphere configuration was ≈1–$1.4\times R_{core}$ (ideally, the largest size in this range is the preferred option because it holds a larger drug payload) and 1×ΔR. The trade-off with this design is that the microspheres had released less than 90% of their load by the time that they fell below the therapeutic release rate threshold.

If we instead consider the therapeutic drug release rate threshold to be 1 μg/day, then the intersection of the time to reach the cumulative drug release and drug release rate thresholds (Figure S11c,d in the Supplementary Materials) occurs around 180 days for a chitosan–PCL bi-layered microsphere configuration with $\approx 0.95\times R_{core}$ and 1×ΔR. The time above the drug release rate threshold reaches a broad maximum region (Figure S11d in the Supplementary Materials) around 220 days at ≈1.4–$1.9\times R_{core}$ and 1×ΔR. While these configurations do not reach 90% cumulative release, they exceed 75% release by this time (Figure S9 in the Supplementary Materials).

**Figure 8:**
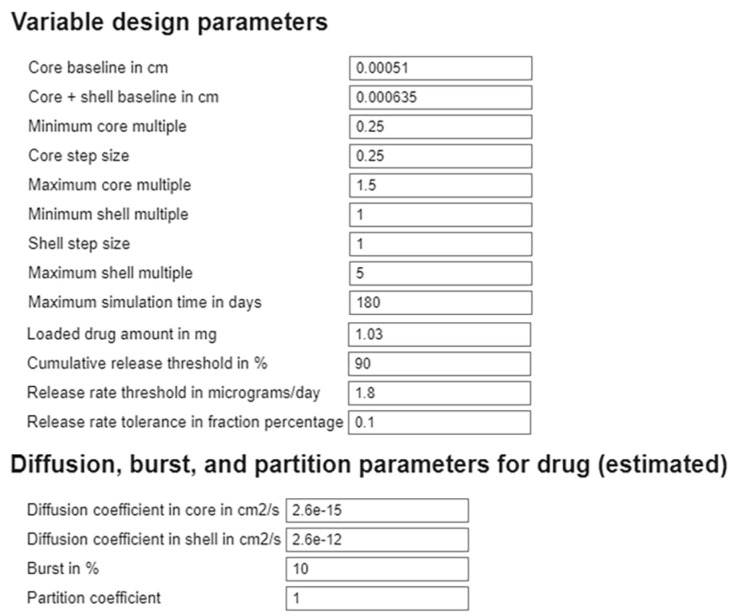
Script screenshot of the MATLAB-based simulation tool for core–shell drug delivery systems (DDSs). Users can specify baseline size values, the range of multipliers for those baseline values, thresholds, and tolerances. The tool also allows modification of estimated transport parameters. The output consists of a drug cumulative release plot and a drug release rate plot for all combinations of core and shell dimensions, facilitating the design and optimization of long-acting DDSs.

To facilitate the design and analysis of core–shell drug delivery systems, we developed an interactive MATLAB live script that enables rapid exploration of how the dimensions of the polymer layers and transport parameters influence the dynamics of drug release (Figure 8). The tool accepts user-defined inputs for the baseline core radius and shell thickness, along with the minimum, maximum, and step sizes for the multipliers used to scale these baseline values. Additional input parameters include maximum simulation time, thresholds for both cumulative drug release and daily release rate, and a tolerance range around the release rate threshold to allow for flexible performance assessments. Users can also modify the values of previously estimated parameters, including drug diffusion coefficients, the partition coefficient, and burst release, to examine the impacts of these parameters on release profiles. The tool generates plots corresponding to several possible combinations of core and shell size multipliers within the specified range. Each plot displays either cumulative release (similar to Figure 5) or the release rate (similar to Figure 6) as a function of time, enabling visualizations of how drug delivery performance changes with geometry specifications. This flexible and user-friendly framework allows researchers to efficiently assess design trade-offs and optimize core–shell configurations for long-term therapeutic delivery.

## 4. Discussion

In this work, we combined mathematical modeling, numerical simulations, and sensitivity-guided parameter estimation into a computational framework tailored for chitosan–PCL bi-layered microspheres for intravitreal controlled release to treat chronic retinal diseases. We developed and parameterized a diffusion-controlled model to characterize long-term drug release from core–shell microspheres, focusing on intravitreal therapies for chronic diseases like wet AMD, where repeated injections present challenges for patient compliance and safety. Using experimental release data for BSA and bevacizumab, we estimated key transport parameters (drug diffusion coefficients, the partition coefficient, and the burst release) using both finite difference (MATLAB) and finite element (COMSOL) implementations.

While prior models [48,49] have applied similar frameworks to glucose and metronidazole release from multi-layered systems, they often made simplifying assumptions involving elements such as unity partition coefficients, no burst release, and short delivery time frames. In contrast, our model explicitly incorporates burst release and partitioning effects (albeit with this effect optimized to a value of 1), extending the analysis to clinically relevant long-term delivery periods and offering a more comprehensive representation of ocular drug transport from bi-layered spherical DDSs.

We verified our computational framework through strong agreement between MATLAB and COMSOL implementations (Figure 3 and Figure 4 and Figures S2 and S3 in the Supplementary Materials). The 50 multi-start parameter estimations in MATLAB (using parallel parfor loops) required 12–16 days to complete on a Dell Precision 3650 Tower Windows PC with 32 GB RAM and an 11th-generation Intel core i7-11700 processor with eight cores, all purchased online in Buffalo, NY, USA. In comparison, COMSOL estimations took around 6 days and required manual input of initial guesses. Despite slight differences in the estimated values for the burst release and the diffusion coefficients of drugs in chitosan (Table 2), both implementations yielded consistent results overall. The MATLAB model was used for design exploration due to its faster runtime for the forward simulation problem (as compared to the inverse parameter estimation problem) and better support for automation. In contrast, COMSOL offers advantages that can be leveraged to deal with more complex geometries where simple approximations are insufficient.

The sensitivity analysis identified three distinct diffusion regimes and quantified how each parameter influenced the 28-day cumulative drug release (Figure 2). The relative importance of the parameters in the sensitivity analysis depended on the specific regime considered. In the core-limited regime ($D_{core}<<D_{shell}$), changes in core diffusivity had the greatest impact, while, in the shell-limited regime ($D_{core}>>D_{shell}$), diffusivity in the shell was more influential than diffusivity in the core; this is consistent with the findings of Barchiesi et al. [49]. Notably, both local and global sensitivity analyses ranked parameters consistently across the three regimes and showed minimal interaction effects.

To demonstrate the utility of our framework, we applied it to a case study involving chitosan–PCL microspheres loaded with bevacizumab, a widely used anti-VEGF therapeutic. Using clinically relevant thresholds of 2 μg/day [72] for daily delivery and 90% cumulative release [62], we examined how varying the core radius ($R_{core}$) and shell thickness (ΔR) influenced therapeutic performance. While increasing $R_{core}$ or ΔR slowed cumulative drug diffusion (Figure 5 and Figure 7a), an increase in $R_{core}$ from its baseline value had little effect on drug release rate until it exceeded $1.5\times R_{core}$, where the release rate began to decline (Figure 6 and Figure 7b). Conversely, reducing $R_{core}$ accelerated drug release, shortening the duration above the release rate threshold. The increase in ΔR needed to be substantial to observe a slight increase in the number of days the drug release rate stayed above the threshold. This implies that tuning $R_{core}$ is the best way to optimize therapeutic delivery and that ΔR adjustments need to be substantial for meaningful drug release modifications.

For a therapeutic release rate target of 2 μg/day of bevacizumab, the best configuration for sustained therapeutic release and efficient drug depletion was around $0.65\times R_{core}$ and 1×ΔR at 90 days (Figure 7c,d). The alternative best configuration that maximized the time above the therapeutic release rate threshold was around $1.4\times R_{core}$ and 1×ΔR (Figure 7c,d) for 100 days, while leaving some loaded drug to release at a sub-therapeutic daily rate for a longer time. For a reduced therapeutic release rate target of 1 μg/day of bevacizumab, the equivalent best configurations were for $0.95\times R_{core}$ and 1×ΔR at 180 days and $1.9\times R_{core}$ and 1×ΔR for 220 days (Figure S11c,d in the Supplementary Materials). These findings highlight the importance of prioritizing the tuning of the core size in DDS design in the core-limited regime ($D_{core}<<D_{shell}$).

All computational models have their limitations, and validation is still an important next step. A practical approach to validate the current model results would be to assess bevacizumab release from a bi-layered microsphere with a configuration of $0.5\times R_{core}$ and baseline ΔR over a short duration (e.g., 1 month). The experimentally measured release profile should be close to the predicted release (Figure 5) with cumulative release exceeding 75% by 30 days. Moreover, here we only parameterized the models to in vitro data. The design trends should still hold when moving to in vivo, but the actual days to reach the release thresholds will be influenced by the in vivo conditions. Notably, erosion should probably be considered in that case as PCL enzymatic degradation in vivo has been shown to be faster than hydrolytic degradation in vitro [16,73]. We also assumed perfect sink external boundary conditions, where the released drug is instantaneously removed from the DDS surface. The model defined here could be combined with a more realistic treatment of the intravitreal conditions, such as in our recent three-dimensional ocular pharmacokinetics models for rabbit and human eyes [74]. Together, the intravitreal drug release could be validated or re-parameterized using in vivo data as they become available.

Finally, an additional contribution of this work is the development of a generalizable and open computational framework for designing and optimizing diffusion-controlled core–shell DDSs. Our dual implementation in MATLAB and COMSOL enables experimentalists and modelers to estimate drug-specific parameters and validate models against release data, demonstrated here for a relatively simple bi-layered microsphere geometry. The COMSOL code can be easily modified to accommodate more complex geometries. Moreover, our MATLAB design script facilitates the efficient exploration of design trade-offs. The framework of this work supports model-informed DDS design, accelerating the development of long-acting ocular therapeutics. We have shared all of these codes openly at https://github.com/ashleefv/LayeredSpheres_DDSdesign, accessed on 25 July 2025 [75].

## 5. Conclusions

In this study, we developed a diffusion-based computational model to investigate long-acting intravitreal drug delivery using core–shell microspheres. The model accounts for key transport process parameters, including burst release, drug partitioning, and diffusion coefficients across bi-layered polymeric spheres. Sensitivity analyses revealed different parameter dominance depending on the specific parameter regimes driving drug release. In the chitosan–PCL case study, we identified drug diffusivity in the core and burst release as the primary drivers of the long-term release behavior. The estimated transport parameters were fitted to experimental release data for BSA and bevacizumab using both finite difference (MATLAB) and finite element (COMSOL) methods. Using clinically relevant thresholds for therapeutic delivery, we simulated drug release from chitosan–PCL microspheres and showed that the core radius has a greater impact on therapeutic performance than the shell thickness in the data-driven parameter regime. We further developed a customizable MATLAB tool to explore the trade-off of using different layer sizes through automated simulations, providing visual outputs of the profiles of the cumulative drug release and drug release rate across a range of core and shell sizes. This tool supports rapid hypothesis testing and formulation design, and the generalizable modeling framework enables easy adaptation to other bi-layered spherical DDSs. Our findings underscore the importance of mechanistic modeling in informing the design of sustained-release drug delivery systems to enhance the treatment of chronic ocular diseases.

## Figures and Tables

**Table 1:** Limits used in the multi-start parameter estimation. *B*: burst release. $D_{Chi}=D_{core}$: drug diffusion coefficient in the chitosan (Chi) core.

Parameter	Lower Limit	Upper Limit	Units
*B*	0	20	%
$D_{Chi}$	$1\times 10^{-15}$	$1\times 10^{-13}$	cm^2^/s

**Table 2:** Estimated parameters for fitting the model to data for drug-loaded chitosan–PCL core–shell microspheres from Jiang et al. [30]. The average model was obtained by averaging the parameters obtained from all the optimization runs that achieved an error within 5% of the minimum error after 50 multi-start attempts. The best model corresponds to the parameter set with the lowest error value. The other model parameters were fixed as κ=1 and $D_{PCL}=1000\times D_{Chi}$. BSA: bovine serum albumin. κ: partition coefficient. $D_{PCL}=D_{shell}$: drug diffusion coefficient in the polycaprolactone (PCL) shell. *B*: burst release. $D_{Chi}=D_{core}$: drug diffusion coefficient in the chitosan (Chi) core. C.I.: confidence interval.

BSA in MATLAB					
Average Model			Best Model		
Parameter	Value ± 95% C.I.	Units	Parameter	Value	Units
*B*	5±1	%	*B*	4.72	%
$D_{Chi}$	$2.9\times 10^{-15}\pm 2.6\times 10^{-15}$	cm^2^/s	$D_{Chi}$	$2.88\times 10^{-15}$	cm^2^/s
Error value	211.62	-	Error value	209.34	-
**BSA in COMSOL**					
**Average Model**			**Best Model**		
**Parameter**	**Value**	**Units**	**Parameter**	**Value**	**Units**
*B*	4	%	*B*	3.86	%
$D_{Chi}$	$2.9\times 10^{-15}$	cm^2^/s	$D_{Chi}$	$2.91\times 10^{-15}$	cm^2^/s
Error value	207.20	-	Error value	207.10	-
**Bevacizumab in MATLAB**					
**Average Model**			**Best Model**		
**Parameter**	**Value ± 95% C.I.**	**Units**	**Parameter**	**Value**	**Units**
*B*	10±6	%	*B*	10.3	%
$D_{Chi}$	$2.6\times 10^{-15}\pm 1.6\times 10^{-15}$	cm^2^/s	$D_{Chi}$	$2.59\times 10^{-15}$	cm^2^/s
Error value	435.60	-	Error value	435.06	-
**Bevacizumab in COMSOL**					
**Average Model**			**Best Model**		
**Parameter**	**Value**	**Units**	**Parameter**	**Value**	**Units**
*B*	9.6	%	*B*	9.56	%
$D_{Chi}$	$2.6\times 10^{-15}$	cm^2^/s	$D_{Chi}$	$2.59\times 10^{-15}$	cm^2^/s
Error value	440.25	-	Error value	440.09	-

## Data Availability

No new data were created or analyzed in this study. We have provided our code for MATLAB 2024b and COMSOL v6.2 in a repository at https://github.com/ashleefv/LayeredSpheres_DDSdesign, accessed on 25 July 2025 [75].

## Supplementary Materials

The following supporting information can be downloaded at https://www.mdpi.com/article/10.3390/pharmaceutics17091174/s1: File S1. Supplementary Material. This document provides the derivations of the finite difference schemes, details of the model verification and preliminary parameter estimation processes, and additional figures for the spatial concentration distributions, sensitivity analysis, and predictive capabilities extended for twice the duration and half the drug release rate threshold.

## References

[1] Kaur I.P., Kakkar S. (2014). Nanotherapy for posterior eye diseases. J. Control. Release.

[2] Chacin Ruiz E.A., Swindle-Reilly K.E., Ford Versypt A.N. (2023). Experimental and mathematical approaches for drug delivery for the treatment of wet age-related macular degeneration. J. Control. Release.

[3] Mi F.L., Tan Y.C., Liang H.F., Sung H.W. (2002). In vivo biocompatibility and degradability of a novel injectable-chitosan-based implant. Biomaterials.

[4] Cojocariu A., Profire L., Aflori M., Vasile C. (2012). In vitro drug release from chitosan/Cloisite 15A hydrogels. Appl. Clay Sci..

[5] Das S., Chaudhury A., Ng K. (2011). Preparation and evaluation of zinc–pectin–chitosan composite particles for drug delivery to the colon: Role of chitosan in modifying in vitro and in vivo drug release. Int. J. Pharm..

[6] Justin R., Chen B. (2014). Characterisation and drug release performance of biodegradable chitosan–graphene oxide nanocomposites. Carbohydr. Polym..

[7] Zhang W., Chen X., Li P., He Q., Zhou H. (2007). Chitosan and chitosan/ β-cyclodextrin microspheres as sustained-release drug carriers. J. Appl. Polym. Sci..

[8] Zhou J., Zhou L., Chen Z., Sun J., Guo X., Wang H., Zhang X., Liu Z., Liu J., Zhang K. (2025). Remineralization and bacterial inhibition of early enamel caries surfaces by carboxymethyl chitosan lysozyme nanogels loaded with antibacterial drugs. J. Dent..

[9] Nair L., Laurencin C. (2007). Biodegradable polymers as biomaterials. Prog. Polym. Sci..

[10] Astuti S., Rahma W.A., Budianto E. (2020). Biodegradable Microcapsules from D,L–PLA/PCL as Controlled Nifedipine Drug Delivery Carrier. Macromol. Symp..

[11] Gedik B., Erdem M.A. (2025). Electrospun PCL membranes for localized drug delivery and bone regeneration. BMC Biotechnol..

[12] Mirzaeei S., Mansurian M., Asare-Addo K., Nokhodchi A. (2021). Metronidazole- and Amoxicillin-Loaded PLGA and PCL Nanofibers as Potential Drug Delivery Systems for the Treatment of Periodontitis: In Vitro and In Vivo Evaluations. Biomedicines.

[13] Mitxelena-Iribarren O., Riera-Pons M., Pereira S., Calero-Castro F.J., Castillo Tuñón J.M., Padillo-Ruiz J., Mujika M., Arana S. (2023). Drug-loaded PCL electrospun nanofibers as anti-pancreatic cancer drug delivery systems. Polym. Bull..

[14] Tabassi S.A.S., Tekie F.S.M., Hadizadeh F., Rashid R., Khodaverdi E., Mohajeri S.A. (2014). Sustained release drug delivery using supramolecular hydrogels of the triblock copolymer PCL–PEG–PCL and α-cyclodextrin. J. Sol-Gel Sci. Technol..

[15] Allyn M.M., Luo R.H., Hellwarth E.B., Swindle-Reilly K.E. (2022). Considerations for polymers used in ocular drug delivery. Front. Med..

[16] Dias J.R., Sousa A., Augusto A., Bártolo P.J., Granja P.L. (2022). Electrospun polycaprolactone (PCL) degradation: An in vitro and in vivo study. Polymers.

[17] Bartnikowski M., Dargaville T.R., Ivanovski S., Hutmacher D.W. (2019). Degradation mechanisms of polycaprolactone in the context of chemistry, geometry and environment. Prog. Polym. Sci..

[18] Lam C.X.F., Savalani M.M., Teoh S.H., Hutmacher D.W. (2008). Dynamics of in vitro polymer degradation of polycaprolactone-based scaffolds: Accelerated versus simulated physiological conditions. Biomed. Mater..

[19] Badiee P., Varshochian R., Rafiee-Tehrani M., Abedin Dorkoosh F., Khoshayand M.R., Dinarvand R. (2018). Ocular implant containing bevacizumab-loaded chitosan nanoparticles intended for choroidal neovascularization treatment. J. Biomed. Mater. Res. Part A.

[20] Mihailovici R., Croitoriu A., Nedeff F., Nedeff V., Ochiuz L., Vasincu D., Popa O., Agop M., Moraru A., Costin D. (2022). Drug-Loaded Polymeric Particulated Systems for Ophthalmic Drugs Release. Molecules.

[21] Pandit J., Sultana Y., Aqil M. (2021). Chitosan coated nanoparticles for efficient delivery of bevacizumab in the posterior ocular tissues via subconjunctival administration. Carbohydr. Polym..

[22] Ugurlu N., Asik M.D., Çakmak H.B., Tuncer S., Turk M., Çagil N., Denkbas E.B. (2019). Transscleral Delivery of Bevacizumab-Loaded Chitosan Nanoparticles. J. Biomed. Nanotechnol..

[23] Zamboulis A., Nanaki S., Michailidou G., Koumentakou I., Lazaridou M., Ainali N.M., Xanthopoulou E., Bikiaris D.N. (2020). Chitosan and its Derivatives for Ocular Delivery Formulations: Recent Advances and Developments. Polymers.

[24] de Souza S.O.L., Guerra M.C.A., Heneine L.G.D., de Oliveira C.R., Cunha Junior A.D.S., Fialho S.L., Oréfice R.L. (2018). Biodegradable core-shell electrospun nanofibers containing bevacizumab to treat age-related macular degeneration. J. Mater. Sci. Mater. Med..

[25] Iyer S., Lee C., Amiji M.M. (2025). Biodegradable polymeric microsphere formulations of full-length anti-VEGF antibody bevacizumab for sustained intraocular delivery. Drug Deliv. Transl. Res..

[26] Waterkotte T., He X., Wanasathop A., Li S.K., Park Y.C. (2022). Long-Term Antibody Release Polycaprolactone Capsule and the Release Kinetics in Natural and Accelerated Degradation. ACS Biomater. Sci. Eng..

[27] Meng Y., Sun S., Li J., Nan K., Lan B., Jin Y., Chen H., Cheng L. (2014). Sustained release of triamcinolone acetonide from an episcleral plaque of multilayered poly-ε-caprolactone matrix. Acta Biomater..

[28] Alkholief M., Kalam M.A., Raish M., Ansari M.A., Alsaleh N.B., Almomen A., Ali R., Alshamsan A. (2023). Topical Sustained-Release Dexamethasone-Loaded Chitosan Nanoparticles: Assessment of Drug Delivery Efficiency in a Rabbit Model of Endotoxin-Induced Uveitis. Pharmaceutics.

[29] Wu Z., Sun W., Wang C. (2024). Clinical characteristics, treatment, and outcomes of pembrolizumab-induced uveitis. Investig. New Drugs.

[30] Jiang P., Jacobs K.M., Ohr M.P., Swindle-Reilly K.E. (2020). Chitosan-polycaprolactone core-shell microparticles for sustained delivery of bevacizumab. Mol. Pharm..

[31] Jiang P., Chaparro F.J., Cuddington C.T., Palmer A.F., Ohr M.P., Lannutti J.J., Swindle-Reilly K.E. (2020). Injectable biodegradable bi-layered capsule for sustained delivery of bevacizumab in treating wet age-related macular degeneration. J. Control. Release.

[32] Ford Versypt A.N., Pack D.W., Braatz R.D. (2013). Mathematical modeling of drug delivery from autocatalytically degradable PLGA microspheres–A review. J. Control. Release.

[33] Kanjickal D.G., Lopina S.T. (2004). Modeling of drug release from polymeric delivery systems–a review. Crit. Rev. Ther. Drug Carr. Syst..

[34] Siepmann J., Siepmann F. (2008). Mathematical modeling of drug delivery. Int. J. Pharm..

[35] Baker R. (1987). Controlled Release of Biologically Active Agents.

[36] Higuchi T. (1961). Rate of release of medicaments from ointment bases containing drugs in suspensions. J. Pharm. Sci..

[37] Roseman T.J., Higuchi W.I. (1970). Release of medroxyprogesterone acetate from a silicone polymer. J. Pharm. Sci..

[38] Roseman T.J. (1972). Release of steroids from a silicone polymer. J. Pharm. Sci..

[39] Crank J. (1975). The Mathematics of Diffusion.

[40] Vergnaud J. (1993). Controlled Drug Release of Oral Dosage Forms.

[41] Lu S.M., Chen S.R. (1993). Mathematical analysis of drug release from a coated particle. J. Control. Release.

[42] Hadjitheodorou A., Kalosakas G. (2014). Analytical and numerical study of diffusion-controlled drug release from composite spherical matrices. Mater. Sci. Eng. C.

[43] Kaoui B., Lauricella M., Pontrelli G. (2018). Mechanistic modelling of drug release from multi-layer capsules. Comput. Biol. Med..

[44] Carr E.J., Pontrelli G. (2018). Modelling mass diffusion for a multi-layer sphere immersed in a semi-infinite medium: Application to drug delivery. Math. Biosci..

[45] Wang S., Lou X. (2010). Numerical methods for the estimation of effective diffusion coefficients of 2D controlled drug delivery systems. Optim. Eng..

[46] Wang S., Mahali S.M., McGuiness A., Lou X. (2010). Mathematical models for estimating effective diffusion parameters of spherical drug delivery devices. Theor. Chem. Acc..

[47] Mohd-Mahali S., Wang S., Lou X., Pintowantoro S. (2012). Numerical methods for estimating effective diffusion coefficients of three-dimensional drug delivery systems. Numer. Algebra Control Optim..

[48] Bielinski C., Kaoui B. (2021). Numerical method to characterise capsule membrane permeability for controlled drug delivery. Int. J. Numer. Methods Biomed. Eng..

[49] Barchiesi E., Wareing T., Desmond L., Phan A.N., Gentile P., Pontrelli G. (2022). Characterization of the shells in layer-by-layer nanofunctionalized particles: A computational study. Front. Bioeng. Biotechnol..

[50] Morton K.W., Mayers D.F. (2005). Numerical Solution of Partial Differential Equations.

[51] LeVeque R.J. (2007). Finite Difference Methods for Ordinary and Partial Differential Equations: Steady-State and Time-Dependent Problems.

[52] Axelsson O., Barker V.A. (2001). Finite Element Solution of Boundary Value Problems: Theory and Computation.

[53] Dorfman K.D., Daoutidis P. (2017). Numerical Methods with Chemical Engineering Applications.

[54] Carslaw H.S., Jaeger J.C. (1986). Conduction of Heat in Solids.

[55] Ozisik M.N. (1993). Heat Conduction.

[56] Ford Versypt A.N., Braatz R.D. (2014). Analysis of finite difference discretization schemes for diffusion in spheres with variable diffusivity. Comput. Chem. Eng..

[57] Tosa V., Mercea P. (2008). Solution of the diffusion equation for multilayer packaging. Plastic Packaging: Interactions with Food and Pharmaceuticals.

[58] Sundqvist H., Veronis G. (1970). A simple finite-difference grid with non-constant intervals. Tellus.

[59] Kalnay de Rivas E. (1972). On the Use of Nonuniform Grids in Finite-Difference Equations. J. Comput. Phys..

[60] Garcia D. (2025). Simpson’s Rule for Numerical Integration. MATLAB Central File Exchange.

[61] Moreno E., Larese A., Cervera M. (2016). Modelling of Bingham and Herschel–Bulkley flows with mixed P1/P1 finite elements stabilized with orthogonal subgrid scale. J. Non-Newton. Fluid Mech..

[62] Van Kampen E., Vandervelden C., Fakhari A., Qian J., Berkland C., Gehrke S.H. (2018). Design of Hollow Hyaluronic Acid Cylinders for Sustained Intravitreal Protein Delivery. J. Pharm. Sci..

[63] Sobol’ I.M. (1990). Sensitivity estimates for nonlinear mathematical models. Mat. Model..

[64] Saltelli A. (2002). Making best use of model valuations to compute sensitivity indices. Comput. Phys. Commun..

[65] Saltelli A., Ratto M., Andres T., Campolongo F., Cariboni J., Gatelli D., Saisana M., Tarantola S. (2008). Variance-based methods. Global Sensitivity Analysis. The Primer.

[66] Morris M.D. (1991). Factorial sampling plans for preliminary computational experiments. Technometrics.

[67] Campolongo F., Cariboni J., Saltelli A. (2007). An effective screening design for sensitivity analysis of large models. Environ. Model. Softw..

[68] Saltelli A., Ratto M., Andres T., Campolongo F., Cariboni J., Gatelli D., Saisana M., Tarantola S. (2008). Elementary effects method. Global Sensitivity Analysis. The Primer.

[69] COMSOL (2021). Uncertainty Quantification Module Users Guide. https://doc.comsol.com/6.0/doc/com.comsol.help.uq/UncertaintyQuantificationModuleUsersGuide.pdf.

[70] Beck J., Arnold K. (1977). Matrix analysis for linear parameter estimation. Parameter Estimation in Engineering and Science.

[71] Avery R.L., Pearlman J., Pieramici D.J., Rabena M.D., Castellarin A.A., Nasir M.A., Giust M.J., Wendel R., Patel A. (2006). Intravitreal bevacizumab (Avastin) in the treatment of proliferative diabetic retinopathy. Ophthalmology.

[72] Carichino L., Guidoboni G., Kansara V., Ciulla T., Harris A., Wanduku D., Zheng S., Zhou H., Chen Z., Sills A., Agyingi E. (2024). Gene therapy bio-factory: Mathematical modeling of the human eye pharmacokinetics. Applied Mathematical Analysis and Computations II.

[73] Bölgen N., Menceloğlu Y.Z., Acatay K., Vargel I., Pişkin E. (2005). In vitro and in vivo degradation of non-woven materials made of poly(*ϵ*-caprolactone) nanofibers prepared by electrospinning under different conditions. J. Biomater. Sci. Polym. Ed..

[74] Chowdhury J.M., Chacin Ruiz E.A., Ohr M.P., Swindle-Reilly K.E., Ford Versypt A.N. (2025). Computer modeling of bevacizumab drug distribution after intravitreal injection in rabbit and human eyes. J. Pharm. Sci..

[75] Chacin Ruiz E.A., Carpenter S.L., Swindle-Reilly K.E., Ford Versypt A.N. (2025). LayeredSpheres_DDSdesign. https://github.com/ashleefv/LayeredSpheres_DDSdesign.

